# Supplementary material for: MetaRibo-Seq measures translation in microbiomes
Source: Nat Commun. 2020 Jun 29;11:3268. doi: 10.1038/s41467-020-17081-z (PMC7324362; doi:10.1038/s41467-020-17081-z)
Supplement: Supplementary file 10 — Supplementary Data 7 [file 41467_2020_17081_MOESM10_ESM.zip › File2/Confidence_High_Taxonomy/221318_out.krona.html]

Javascript must be enabled to view this page.

members
magnitude
magnitudeUnassigned
count
unassigned
taxon
rank

221318\_out

5

superkingdom
5
2

5
1239
phylum

5
186801
class

186802
5
order

541000
5
family

genus
5
216851


SRS011302\_contig\_number\_18771SRS015431\_contig\_number\_81005SRS019068\_contig\_number\_39384SRS078242\_contig\_number\_6503SRS1041157\_contig\_number\_4657
853
5
species
